# The Oropharyngeal Airway in Young Adults with Skeletal Class II and Class III Deformities: A 3-D Morphometric Analysis

**DOI:** 10.1371/journal.pone.0148086

**Published:** 2016-02-22

**Authors:** Yasas Shri Nalaka Jayaratne, Roger Arthur Zwahlen

**Affiliations:** 1 Division of Orthodontics, Dept. of Craniofacial Sciences, University of Connecticut School of Dental Medicine, Farmington, Connecticut, United States of America; 2 Discipline of Oral & Maxillofacial Surgery, Faculty of Dentistry, The University of Hong Kong, Sai Ying Pun, Hong Kong Special Administrative Region, Peoples Republic of China; Georgia Regents University, College of Dental Medicine, UNITED STATES

## Abstract

**Objectives:**

1) To determine the accuracy and reliability of an automated anthropometric measurement software for the oropharyngeal airway and 2) To compare the anthropometric dimensions of the oropharyngeal airway in skeletal class II and III deformity patients.

**Methods:**

Cone-beam CT (CBCT) scans of 62 patients with skeletal class II or III deformities were used for this study. Volumetric, linear and surface area measurements retroglossal (RG) and retropalatal (RP) compartments of the oropharyngeal airway was measured with the 3dMDVultus software. Accuracy of automated anthropometric pharyngeal airway measurements was assessed using an airway phantom.

**Results:**

The software was found to be reasonably accurate for measuring dimensions of air passages. The total oropharyngeal volume was significantly greater in the skeletal class III deformity group (16.7 ± 9.04 mm^3^) compared with class II subjects (11.87 ± 4.01 mm^3^). The average surface area of both the RG and RP compartments were significantly larger in the class III deformity group. The most constricted area in the RG and RP airway was significantly larger in individuals with skeletal class III deformity. The anterior-posterior (AP) length of this constriction was significantly greater in skeletal class III individuals in both compartments, whereas the width of the constriction was not significantly different between the two groups in both compartments. The RP compartment was larger but less uniform than the RG compartment in both skeletal deformities.

**Conclusion:**

Significant differences were observed in morphological characteristics of the oropharyngeal airway in individuals with skeletal class II and III deformities. This information may be valuable for surgeons in orthognathic treatment planning, especially for mandibular setback surgery that might compromise the oropharyngeal patency.

## Introduction

The availability of modern imaging tools enables the assessment of static and dynamic changes in the structure and function of the upper pharyngeal space. The main imaging modalities used for these purposes include nasopharyngoscopy, acoustic reflection, fluoroscopy, lateral cephalometry, Multislice Computerized Tomography (MSCT) and Magnetic Resonance Imaging (MRI). Nasopharyngoscopy facilitates the evaluation of the pharyngeal lumen, but not the surrounding soft tissues. Furthermore, patients can be uncooperative due to the invasive nature of this procedure. Although acoustic reflection (echo imaging) can be used for calculating the area of the upper pharyngeal space as a function of the distance from the mouth, it fails to provide high resolution images**[1,2]**.

Lateral cephalometry has been used in the past for airway analysis because it is wide available and relatively inexpensive. Nevertheless, magnification and positioning errors, superimposition of midline and lateral structures, problems in identifying soft-tissue contours are some of the limitation of this technique. In addition, lateral cephalometry fails to provide any information about the transverse dimension or volumes of the airway. MRI provides three-dimensional representation of the upper airway and surrounding soft tissues without radiation exposure. However, MRI is not routinely used for airway assessment due to high costs and the space required to house these units.

The introduction of cone-beam computed tomography (CBCT) has significantly popularized 3-D imaging in the maxillofacial region. In contrast to multi-slice computed tomography (MSCT) which uses a fan shaped beam to acquire images as multiple slices, CBCT uses a single cone shaped beam to gather the full volume of image data in a single rotation. The main benefit of CBCT over conventional CT scanning is the marked reduction in the radiation dose. In contrast to MSCT, it is not possible to clearly differentiate various soft tissue structures with CBCT. Nevertheless, CBCT can be used for evaluating the oropharyngeal airway because it demonstrates the interface between the soft tissue and air with a high spatial resolution[3].

The soft tissue lining the oropharyngeal airway is attached to bony components that are manipulated during orthognathic surgery. Alterations to this osseous framework will lead to postoperative changes in the dimensions of the pharyngeal airway[4].Therefore, detailed information on anthropometric characteristics of the pharyngeal airway in common dentofacial deformities is of value when planning orthognathic surgery.

In the past, the majority of studies on the pharyngeal airway have been conducted with 2-D cephalometry. Most of the 3-D studies have focused on obstructive sleep apnoea (OSA) or pharyngeal morphology of children and adolescents. However, there is a dearth of scientific data on characteristics of the pharyngeal airway in adult subjects without OSA.

The skeletal patterns in class II and III deformities depend on the relationship between the maxilla and mandible. The skeletal class II deformity may arise from either deficient mandibular growth, excessive maxillary growth or a combination of both. Similarly, a skeletal class III deformity results from a prognathic mandible, a retrognathic maxilla or due to both these reasons. The dimensions of the retropalatal oropharyngeal compartment can be determined by the soft palate which is attached to the maxilla, whereas the retroglossal compartment is related to the tongue which is attached to the mandible and hyoid bone. A separate anthropometric analysis of these two compartments can reveal interesting findings which may be clinically relevant when planning orthognathic surgery. Therefore, the objectives of this study were1) to determine the accuracy and reliability of an automated anthropometric measurement software for the oropharyngeal airway, and2) to compare the anthropometric dimensions of oropharyngeal airways in skeletal class II and III deformity patients.

## Materials and Methods

### Ethics Statement

This study was approved by the Institutional Review Board (IRB) of the University of Hong Kong/ Hospital Authority Hong Kong West Cluster (Protocol No: UW 12–066). As these CBCT scans were acquired as a part of the routine treatment protocol for orthognathic treatment planning and due to the retrospective nature of this study, a waiver of consent was granted by the IRB.

### Subjects

CBCT scans of patients with skeletal class II and III deformities referred to the Discipline of Oral & Maxillofacial Surgery, Prince Philip Dental Hospital have been used for this study. Patients with an obvious facial asymmetry, a history of OSA, congenital deformities such as cleft lip/palate, syndromes, trauma or previous maxillofacial surgery were excluded. The reference values used for classifying subjects into skeletal class II or III deformity groups can be found in S1 Appendix. The method of identifying these landmarks and computing these measurements have been explained in standard cephalometric atlases [5–7]. Subjects meeting at least three out of these four criteria were classified under the Class II or III facial deformity type:

1. SNB angle
2. ANB angle
3. Incisor overjet
4. Wits appraisal

Each subject had a CBCT scan (i-CAT System, Imaging Sciences International, Hatfield, PA, USA) with the full field of view (16cm x 13cm) or extended field of view (16cm x 22cm) depending on the facial height. The subjects were instructed to remain still while breathing normally through the nose. The chin cap was not used as it can distort soft tissues. Each scan was stored in the Digital Imaging and Communications in Medicine (DICOM) format for further processing.

### Image Analysis

The CBCT scans were de-identified and analyzed anonymously. The 3dMDVultus software (3dMD LLC, Atlanta, GA, USA) was used for anthropometric analysis of the oropharyngeal airway. The DICOM images were first loaded on to the software and the orientation of the image was fine-tuned to simulate the natural head position. The layout of the software permitted the visualization of the axial, sagittal and coronal slides as well as the 3-D rendered model in a single screen.

**Fig 1:**
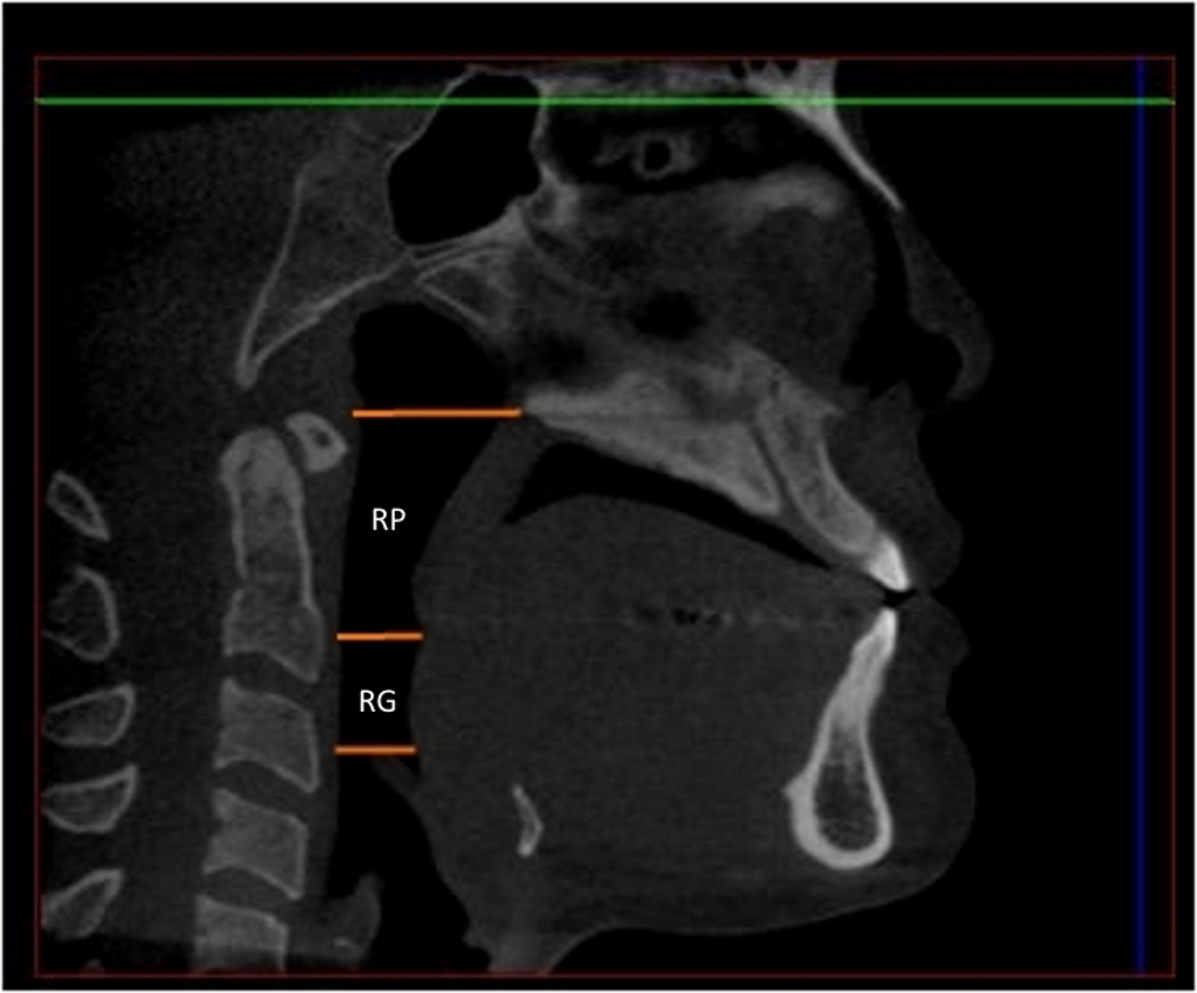
Boundaries of the retropalatal (RP) and retroglossal (RG) compartments.

The oropharyngeal airway was first outlined using the airway extraction tool available in the software. Horizontal planes parallel to the SN line according to the definitions by Zhao *et al*[8]were used as the superior and inferior boundaries of the oropharyngeal compartment. The superior boundary of the oropharynx was defined as the horizontal plane through the posterior nasal spine. The inferior boundary was defined as the plane passing through the most superior point of the epiglottis. A horizontal plane through the tip of the uvula divided the oropharynx into retroglossal (RG) and retropalatal (RP) compartments (Fig 1).

The shape of the extraction path was adjusted by moving the control points highlighted as yellow dots in Fig 1. The width and breath of the extraction path were modified to ensure that it encompasses all the boundaries of the oropharyngeal airway. Once the extraction path was fine-tuned the software was able to generate cross sectional images of the oropharyngeal airway and automatically calculate the total volume. In addition, it indicated the anteroposterior width, the transverse width and the total cross-sectional area of each slice. The lengths of the RP and RG airway compartments were measured on the mid sagittal CBCT slice using the boundaries stated above. The following primary anthropometric measurements were recorded for each of these compartments as per Abramson *et al*[9]:

1. Vertical length
2. Volume
3. Minimum surface area
4. Anteroposterior (AP) length of the most constricted area
5. Transverse width of the most constricted area

**Fig 2:**
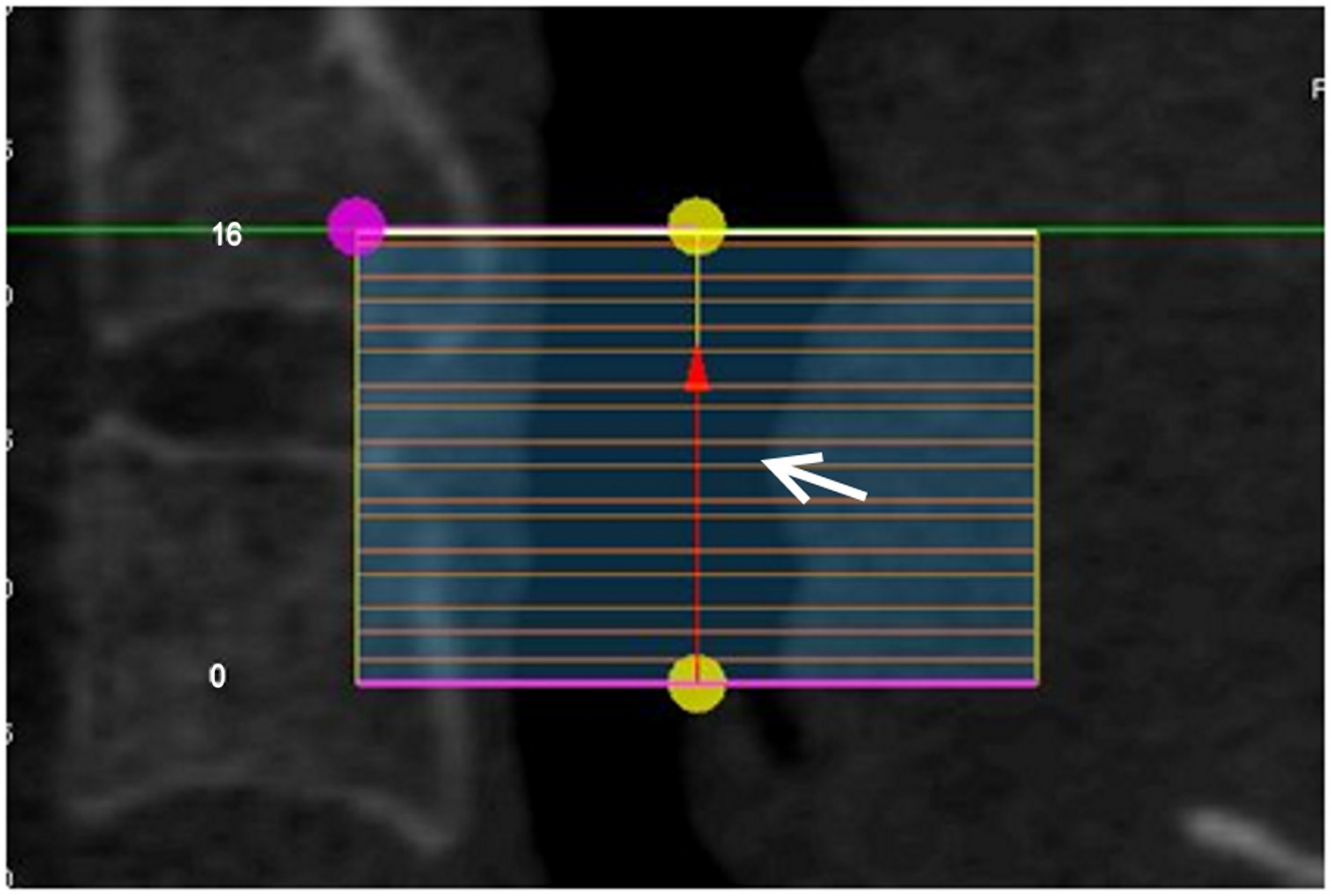
An example illustrating the method of recording the location of the most constricted region (white arrow).

The level of the most constricted area—this location was recorded as a fraction of the total vertical length of the compartment measured from its inferior border, e.g. if the overall RG compartment illustrated in Fig 2 consisted of sixteen 1mm slices and the most constricted region was located in the 7^th^ slice (counted from the bottom), the level of the constriction is 7/16 = 0.44.

The following secondary anthropometric measurements and ratios[9] were calculated for each compartment based on primary anthropometric measurements.

1. Average cross sectional area [= volume/ length of the compartment]
2. Ratio between the transverse and AP diameter of the most constricted airway slice
3. Airway uniformity [= minimum cross sectional area/ Average cross sectional area]

### Validation Study

**Fig 3:**
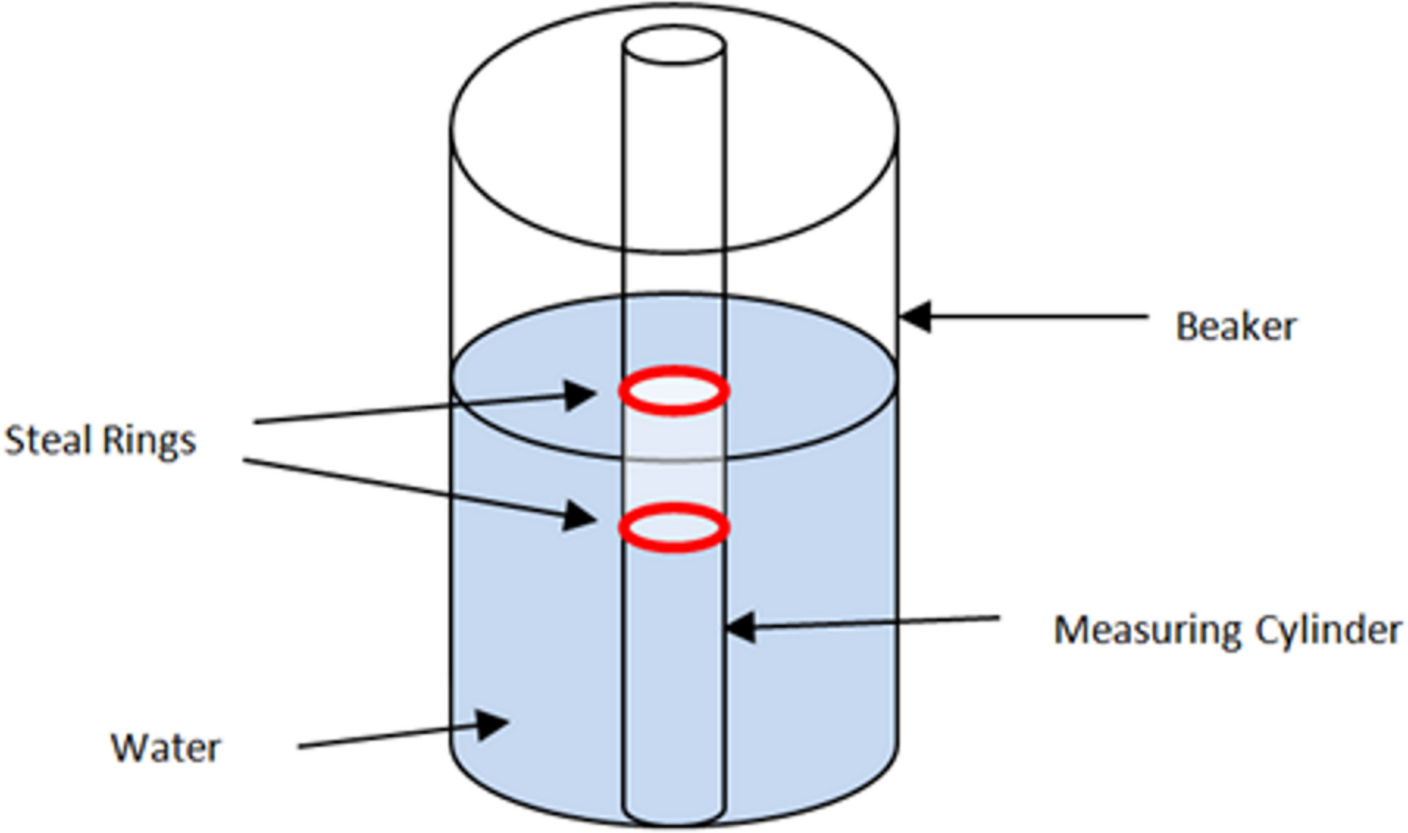
A diagrammatic sketch of the phantom.

**Fig 4:**
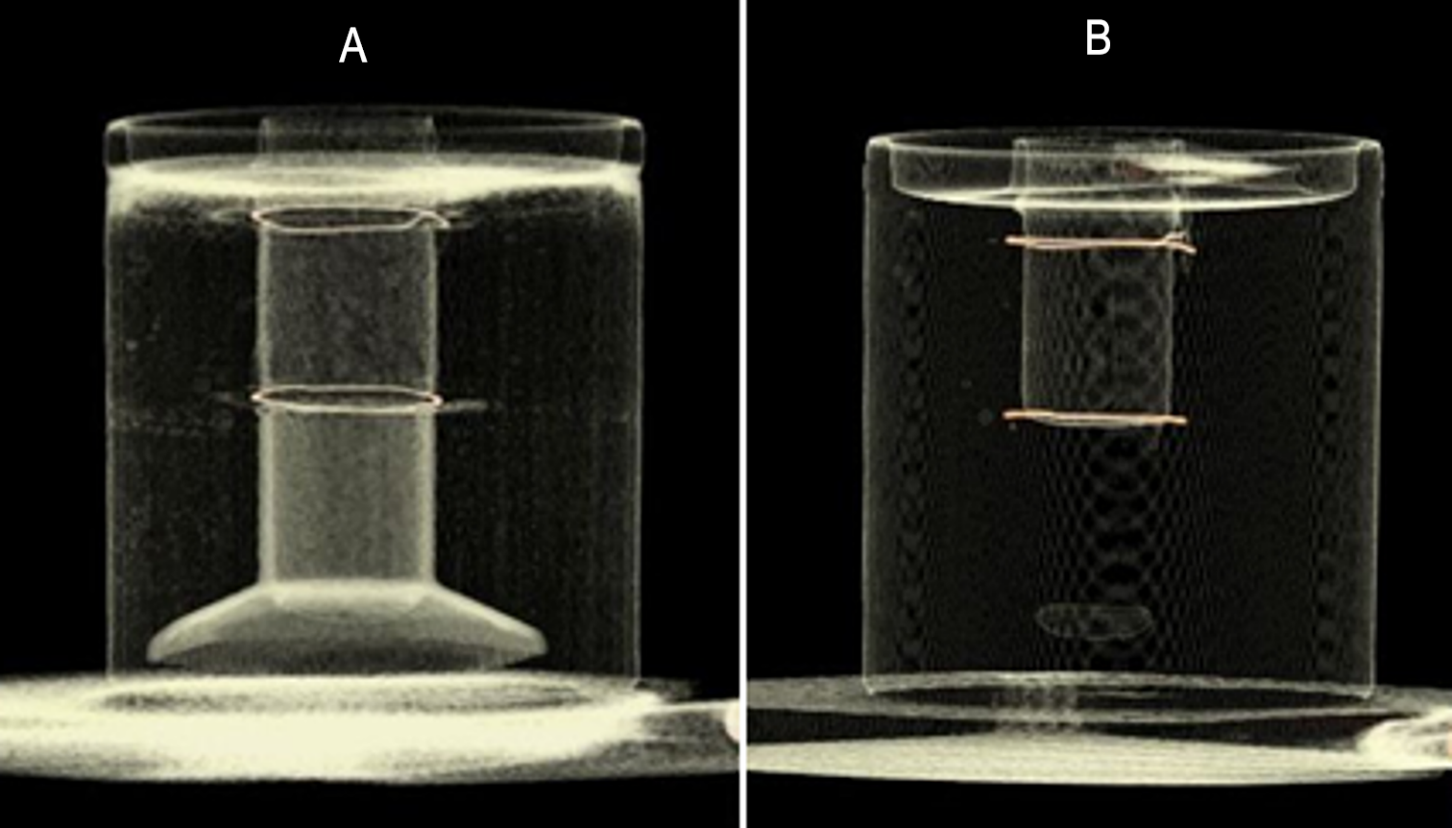
A volume rendered Cone-beam CT image of the phantom. (A) High contrast view (B) Low contrast view.

In order to determine the accuracy of automated anthropometric pharyngeal airway measurements, a phantom depicted in Fig 3 was constructed. This consisted of a plastic measuring cylinder with the volume measurements marked on its surface by the manufacturer at 1 ml increments. Two steel rings were placed between 1 ml markings as it would be visible on a CBCT scan. The whole apparatus was submerged in a plastic beaker containing water. Water was added up to the level of the lower ring to prevent the measuring cylinder from floating. Water was selected as the intervening medium because its attenuation coefficient is closest to soft tissues[10]. A CBCT scan of the whole apparatus was performed (Figs 3 and 4) using the same parameters used for normal subjects. It was hypothesized that the automated anthropometric measurement software should be able to calculate the 1 ml (= 10mm^3^) of air entrapped between the steel rings and the 20mm diameter of the cylinder with reasonable accuracy. Measurements were performed 4 times on separate sessions using the same CBCT dataset, as described in the previous section. The mean volume and the diameter were calculated from these individual measurements.

### Error Analysis

A randomly selected sample of 9 CBCT images were re-measured under the same conditions at least three weeks after the initial measurement session. The technical error of measurement (TEM) was calculated using Dahlberg's formula[11].

### Statistical Analysis

The Shapiro-Wilk test was first performed to test for normality of the data. Based on the outcome of this test, the Mann-Whitney U test was used to compare the distribution of variables between the skeletal class II and III deformity groups. The Wilcoxon signed ranks test was used to compare the paired measurements between RG and RP compartments. These statistical tests were performed with PASW 18 software (SPSS, Chicago, IL, USA).

## Results

### The Validation Study

The average volume of air measured by the software was 9.26 mm^3^. This was quite close to the 10 mm^3^ of air trapped between the steel rings of the measurement cylinder. The mean diameter of the cylinder calculated by the software was 20.79 mm. This was similar to the 20.47 mm actual diameter of the cylinder measured by a Vernier caliper. Thus, the 3dMD Vultus software was considered to be reasonably accurate for measuring dimensions of air passages.

### Assessment of the Oropharyngeal Airway in Patients

CBCT scans of 62 patients (28 males and 34 females) met the inclusion criteria. The skeletal class II group consisted of 27 patients (mean age = 24.7 ± 7.21 years) while the skeletal class III group included 35 patients (mean age = 20.7 ± 3.57 years). There were no statistically significant differences between the two groups in terms of age (p = 0.055).

No significant differences were found between the two groups in relation to the length of the oropharyngeal airway in RG or RP compartment (Tables 1and 2). However, significant differences were observed between individuals with skeletal class II and III deformities, with the latter having higher RP and RG volumes. Consequently, the total oropharyngeal volume was also significantly greater (p = 0.006) in skeletal class III (16.7 ± 9.04 mm^3^) compared to class II individuals (11.87 ± 4.01 mm^3^). The average surface area of both the RG and RP compartments were significantly larger in the Class III group.

**Table 1:** Anthropometric measurements of the retroglossal compartment.

Anthropometric measurement	Class II Deformity					Class III Deformity					P-value
	Mean	SD	Percentiles			Mean	SD	Percentiles			
			25	50	75			25	50	75	
**RG Airway length (mm)**	21.47	7.13	17.03	21.84	27.24	20.99	6.61	15.03	21.05	25.77	0.793
**RG Volume (mm**^**3**^**)**	4.31	2.33	2.77	4.29	5.59	6.26	4.56	3.53	5.25	6.96	0.048*
**Average RG cross sectional area (mm**^**2**^**)**	221.05	184.06	155.37	200.89	243.58	291.77	154.32	198.00	247.06	360.03	0.009*
**Level of most constricted RG area**	0.51	0.40	0.00	0.67	1.00	0.48	0.44	0.00	0.57	1.00	0.860
**RG Minimum cross sectional area (mm**^**2**^**)**	186.21	64.18	134.24	180.96	236.24	276.4	116.17	206.40	253.76	367.05	< 0.001*
**Width of the most constricted RG area (mm)**	25.99	6.00	21.40	25.20	30.40	27.68	5.02	23.60	28.40	31.20	0.324
**AP length of the most constricted RG area (mm)**	8.01	2.91	6.00	8.00	9.60	11.63	4.00	8.80	11.60	13.80	< 0.001*
**Width: length ratio of the most constricted RG area**	3.78	1.99	2.04	3.43	4.81	2.70	1.22	1.83	2.45	2.95	0.015*
**RG Airway Uniformity**	1.00	0.37	0.80	1.00	1.15	0.99	0.16	0.91	1.00	1.09	0.999

* Statistically significant, AP—Anteroposterior, RG- Retroglossal

**Table 2:** Anthropometric measurements of the retropalatal compartment.

Measurement	Class II Deformity					Class III Deformity					*p*- value
	Mean	SD	Percentiles			Mean	SD	Percentiles			
			25	50	75			25	50	75	
**RP Airway length (mm)**	29.11	4.25	26.24	30.00	31.68	30.52	4.02	27.26	30.64	34.01	0.307
**RP Volume (mm**^**3**^**)**	7.56	2.96	5.20	6.98	8.48	10.44	4.97	7.83	9.50	12.73	0.005*
**Average RP cross sectional area (mm**^**2**^**)**	260.59	92.89	193.29	234.43	306.21	345.27	173.64	243.28	302.39	413.08	0.020*
**Level of most constricted RP area**	0.19	0.23	0.00	0.17	0.29	0.31	0.33	0.00	0.20	0.69	0.263
**RP Minimum cross sectional area (mm**^**2**^**)**	211.17	81.44	155.44	190.40	272.96	304.53	166.03	213.68	271.68	333.93	0.010*
**Width of the most constricted RP area (mm)**	25.88	5.62	22.60	25.20	29.60	29.26	7.27	24.20	28.40	32.20	0.098
**AP length of the most constricted RP area (mm)**	8.36	3.32	5.80	8.40	10.00	11.66	4.77	8.80	11.20	14.20	0.002*
**Width: length ratio of the most constricted RP area**	3.57	1.75	2.39	3.11	4.08	2.73	0.83	2.13	2.41	2.89	0.018*
**RP Airway Uniformity**	0.82	0.22	0.76	0.81	0.97	0.88	0.15	0.81	0.89	0.95	0.222

* Statistically significant, AP- Anteroposterior, RP- Retropalatal

The most constricted area in the RG and RP airway was significantly larger in Class III group. No statistically significant differences were found between the 2 groups in relation to the level of the most constricted region, either in the retroglossal (p = 0.860) or retropalatal (p = 0.263) compartments (Tables 1 and 2). The AP length of this constriction was significantly greater in Class III group in both compartments, whereas the width of the constriction was not significantly different between the two groups in both compartments. The oropharyngeal uniformity did not significantly differ among the two groups.

### Comparison between the Retroglossal and Retropalatal Compartments

Length, volume and the average cross sectional area of the RP compartment were significantly greater than in the RG component in both deformity classes (Table 3). In both groups the RG compartment was much more uniform than the RP compartment. In skeletal class II deformity group the level of the most constricted region was located midway in the RG compartment, while it was more caudally placed in the RP compartment. No significant differences were found between the level of the most constricted are in RG versus RP compartments in skeletal class III deformity group.

**Table 3:** Comparison between the two oropharyngeal compartments.

Anthropometric measurement	Class II Deformity			Class III Deformity		
	RG Mean	RP Mean	p- value	RG Mean	RP Mean	p- value
**Airway length (mm)**	21.47	29.11	< 0.001*	20.99	30.52	< 0.001*
**Volume (mm**^**3**^**)**	4.31	7.56	< 0.001*	6.26	10.44	< 0.001*
**Average cross sectional area (mm**^**2**^**)**	221.05	260.59	0.005*	291.77	345.27	0.001*
**Level of most constricted area**	0.51	0.19	0.004*	0.48	0.31	0.104
**Minimum cross sectional area (mm**^**2**^**)**	186.21	211.17	0.079	276.4	304.53	0.140
**Width of the most constricted area (mm)**	25.99	25.88	0.895	27.68	29.26	0.161
**AP length of the most constricted area (mm)**	8.01	8.36	0.753	11.63	11.66	0.533
**Airway Uniformity**	1.00	0.82	0.047*	0.99	0.88	0.004*

* Statistically significant, AP- Anteroposterior, RP- Retropalatal, RG—Retroglossal

### Sexual Dimorphism

There was no sexual dimorphism in relation to any of the measurements in the Class II group. However, in the Class III group, three measurements in the RG compartment (i.e. RG volume, minimum and average cross sectional area) were found to be significantly greater in males (Table 4).

**Table 4:** Gender differences in oropharyngeal parameters.

Parameter	Class II Deformity					Class III Deformity				
	Male (N = 10)		Female (N = 17)		P-value	Male (N = 18)		Female (N = 17)		P-value
	Mean	SD	Mean	SD		Mean	SD	Mean	SD	
**Age**	24.90	8.70	24.53	6.47	0.801	21.17	3.22	20.24	3.95	0.370
**RP Space length (mm)**	31.13	3.07	27.92	4.47	0.098	31.53	4.49	29.44	3.23	0.146
**RG Space length (mm)**	24.44	6.74	19.72	6.95	0.145	22.45	7.73	19.44	4.94	0.210
**RG Volume (mm3)**	4.89	1.93	3.97	2.53	0.132	7.70	5.46	4.75	2.79	0.036*
**Level of most constricted RG area**	0.54	0.37	0.49	0.43	0.777	0.51	0.46	0.46	0.43	0.616
**RG Minimum cross sectional area (mm2)**	186.27	78.35	186.17	56.93	0.744	314.47	136.41	236.09	74.48	0.038*
**Width of the most constricted RG area (mm)**	27.04	6.41	25.36	5.86	0.421	28.64	6.06	26.65	3.52	0.428
**AP length of the most constricted RG area (mm)**	7.80	3.70	8.14	2.45	0.782	12.76	3.93	10.44	3.82	0.105
**RP Volume (mm3)**	7.50	3.11	7.60	2.97	0.802	11.07	5.90	9.78	3.83	0.621
**Level of most constricted RP area**	0.22	0.18	0.18	0.26	0.269	0.35	0.31	0.26	0.34	0.198
**RP Minimum cross sectional area (mm2)**	211.59	91.89	210.93	77.65	0.88	312.58	190.34	296.00	141.18	0.575
**Width of the most constricted RP area (mm)**	25.80	5.96	25.93	5.60	0.744	29.16	8.29	29.36	6.27	0.530
**AP length of the most constricted RP area (mm)**	8.12	3.91	8.49	3.04	0.529	12.00	5.72	11.31	3.64	0.766
**Total Volume (mm3)**	12.40	4.22	11.57	3.98	0.34	18.77	10.78	14.53	6.35	0.187
**Average RG cross sectional area (mm2)**	198.37	59.73	234.40	229.20	0.763	341.82	182.10	238.79	97.85	0.035*
**Average cross sectional RP area (mm2)**	240.47	95.60	272.43	92.09	0.228	355.63	202.73	334.31	141.97	0.817
**Width: length ratio of the most constricted RG area**	4.53	2.83	3.34	1.17	0.451	2.43	0.83	2.98	1.51	0.364
**Width: length ratio of the most constricted RP area**	3.88	2.31	3.38	1.37	0.763	2.67	0.80	2.79	0.88	0.644
**RG Space Uniformity**	0.94	0.25	1.04	0.43	0.498	0.97	0.14	1.02	0.17	0.117
**RP Space Uniformity**	0.87	0.16	0.80	0.24	0.351	0.87	0.12	0.89	0.18	0.766

* Statistically significant, AP- Anteroposterior, RG- Retroglossal, RP- Retropalatal

### Error Analysis

The results of the error analysis are enclosed as S2 Appendix. The technical error of measurement (TEM) for all parameters was within clinically acceptable limits.

## Discussion

There have been several reports on the development of OSA following mandibular setback surgery[12–17]. However, some patients with severe OSA require maxillomandibular advancement to correct this problem. Therefore, information on baseline characteristics of the oropharyngeal airway in common facial skeletal deformities is of value. Areas potentially leading to postoperative airway constriction could therefore be taken into consideration during orthognathic treatment planning.

In the past, the majority of studies on oropharyngeal airway have been conducted with lateral cephalometry. However, this technique has a number of drawbacks including magnification errors, superimposition of midline and lateral structures and difficulty in identifying soft-tissue contours. Furthermore, lateral cephalometry provides only a 2-D profile evaluation without any information on transverse dimensions or pharyngeal volumes. Even though MSCT could be used for 3-D assessment, concerns about radiation exposure limit its routine use.

Most of the CBCT studies on pharyngeal airway have focused on subjects suffering from OSA[3]. As CBCT is a relatively new imaging modality, only few studies on baseline measurements of the oropharyngeal airway in different dentofacial deformities are available. However, in those investigations, the study populations have been primarily children or adolescents[18],[19],[20].

As a part of the treatment protocol, CBCT scans are now being increasingly used for diagnosis and treatment planning for orthodontics or orthognathic surgery. These CBCT scans are an often a neglected resource that can be employed for evaluation of the oropharynx.

### Interpretation of the Main Findings

Iwasaki *et al*.[18] used CBCT to compare the oropharyngeal airway in 20 children with Class III and 25 children with Class I malocclusion. They found that the Class III group had statistically larger surface area with a wider but flatter airway than the Class I group. The pharyngeal area in the Class III group was positively correlated with the severity of the deformity.

Kim *et al.[19]* assessed pharyngeal volume and cross-sectional areas with CBCT in 27 children. They reported that total pharyngeal volume (nasal cavity, nasopharynx and oropharynx) in retrognathic children were significantly smaller than those with a normal skeletal pattern. However, volume measurements in the sub-compartments of the pharynx did not statistically differ between the normal and retrognathic groups. They also noted that pharyngeal volumetric measurements significantly correlated with the ANB angle and anterior facial height.

Tso *et al*.[21] examined the pharyngeal airway in 10 adult Class I subjects. They reported that the most constricted region varies in position but it appears to be primarily in the oropharynx and ranges from 90 to 360 mm^2^. However, the findings of this study cannot be generalized due to the small sample size.

Grauer*et al.[22]*analyzed the total pharyngeal volume in 62 CBCT scans after classifying them into Class I, II and III skeletal pattern. They also divided the same sample into long, short and normal face types based on the facial index. The pharyngeal volume did not correlate with the subject’s age or gender. The volume of Class II subjects in the inferior compartment was significantly smaller, but it did not statistically differ among the short, normal and long face types.

Recently El and Palomo[20] using a sample of adolescents between 14–17 years reported that Class II subjects had a significantly lower oropharyngeal volume than Class I and Class III subjects. These volumes were significantly smaller in individuals with retruded mandibles than those with a high SNB angle. The most constricted region of the oropharynx was located at the level of the tongue base.

We found that subjects with skeletal class III deformity had significantly higher RG, RP total oropharyngeal volumes and average cross-sectional areas. However, the RG and RP compartments between skeletal class II and III deformities did not differ in length. Thus, the volume differences between the two groups have to be mainly attributed to the higher average cross sectional area of the oropharynx in subjects with skeletal class III deformities.

As the mandibular complex is more prognathic and wider in the class III deformities, the soft tissue attached to it could be dragged forward and laterally. This may lead to a widened RG airway and a higher volume. The hypoplastic maxilla which is situated backwards may pull the soft palate in a posterior-superior direction, opening up the RP airway resulting in an overall higher volume in this compartment as well. These findings in individuals with skeletal class III deformities are in line with those of El and Palomo[20]. Likewise, in a recent study Hong *et al*.[23]found higher oropharyngeal volumes in subjects with skeletal class III deformities compared to those with skeletal Class I pattern.

In this study the RG and RP compartments were evaluated separately. Statistical differences were found in both types of deformities indicating that the RP compartment was larger than the RG in terms of length, volume and cross-sectional area. In contrast, the RG compartment was found to be much more uniform in skeletal class II and III deformities.

The AP and lateral dimensions as well as the ratio between these two measurements gives an indication about the shape of the oropharyngeal airway. Even though lateral width of the most constricted regions in the RG as well as RP compartments were not statistically different, the AP dimension was lower in skeletal class II deformities, indicating a flatter oropharyngeal airway. This could be explained by the retrognathic mandible in this type of deformity and concomitant backward thrust of the tongue that might leads to a constriction of the pharyngeal airway

### Significance

3D airway measurements using CBCT provides much more information that could not be acquired from traditional 2D cephalograms. The airway volume, cross sectional areas can only be computed from CBCT scans. Likewise, 2D cephalograms does not provide any information on the transverse dimension of the airway. So far, quantitative 3D baseline data on oropharyngeal morphology Chinese adults were unavailable in the scientific literature. This study has generated contemporary data on the oropharyngeal characteristics in young Chinese adults with skeletal class II and III deformities. This information may be valuable for surgeons when planning orthognathic surgery, especially for mandibular setback surgery.

The subjects of this study did not have previous surgery. Therefore, the influence of orthognathic surgery on oropharyngeal airways did not affect the findings of this study. Selection bias was minimized by including consecutive patients. The accuracy of the 3dMD Vultus software for assessing the oropharyngeal airway was also tested using a phantom. It was found that the software was reasonably accurate in detecting the target volume of air trapped within the phantom.

There are reports in literature about development of OSA following mandibular setback surgery[12–17]. The baseline volume of the RG compartment is smaller than RP in skeletal class III deformities. As the tongue may be pushed backwards with mandibular setback surgery,[24] the RG volume may be more affected than the RP compartment. Furthermore, the RG airway is less uniform than RP at the baseline. As a result, mandibular setback alone may decrease the uniformity further compromising the patency of the RG airway. Therefore, surgeons should be cautious when performing single jaw surgery for patients with skeletal class III deformities in contrast to double jaw surgery, as in the latter, there might be some compensation for the reduction of RG airway by the maxillary advancement which increases the RP airway.

In the skeletal class II deformities, all measurements in the RG compartment were smaller than the RP compartment. As the most constricted region is located almost halfway in the total length of the RG compartment, mandibular advancement alone may enhance the patency of the pharyngeal airway.

### Limitations

It was not possible to include a control group with a skeletal class I pattern in this study due to ethical concerns of exposing those without any facial deformities to radiation.

As the patients included in this study were primarily referred for orthognathic surgery, their oropharyngeal deformity may be considered to be more severe due to the skeletal component than those patients undergoing orthodontic treatment alone due to dentoalveolar deformity only. However, as the subjects recruited are similar to those that normally undergo orthognathic surgery, the baseline data generated from this study is useful for the planning orthognathic surgery of such patients.

In order to explore the relationship of structural information with function, it would be ideal if these CBCT findings could be correlated with findings of polysomnography. Due to cost considerations and the fact that polysomnography requires overnight hospital admission this was not feasible.

## Conclusion

Significant differences were observed in the morphological characteristics of the oropharyngeal airway in individuals with skeletal class II and III deformities. Subjects with class III deformity have significantly larger oropharyngeal volumes and average cross sectional areas than those with skeletal class II deformity. The most constricted region was anteroposteriorly narrower in skeletal class II compared to class III deformities. The retropalatal compartment was larger but less uniform than the retroglossal compartment in both deformity types. This information may be valuable for surgeons in orthognathic treatment planning, especially for mandibular setback surgery that might compromise the oropharyngeal patency.

## Supporting Information

S1 Appendix Cephalometric parameters used for classifying Class II and Class III patients. (DOCX)

S2 Appendix The technical error of measurement (TEM) for different oropharyngeal parameters. (DOCX)

S1 File An Excel file with measurements obtained from each study subject. (XLSX)
