# Supplementary material for: The Oropharyngeal Airway in Young Adults with Skeletal Class II and Class III Deformities: A 3-D Morphometric Analysis
Source: PLoS One. 2016 Feb 22;11(2):e0148086. doi: 10.1371/journal.pone.0148086 (PMC4762707; doi:10.1371/journal.pone.0148086)
Supplement: S1 Appendix — (DOCX) [file pone.0148086.s001.docx]

S1 Appendix. Cephalometric parameters used for classifying Class II and Class III patients

| Measurement | Description | Reference values (Mean ± SD) | |
| --- | --- | --- | --- |
|  |  | Males | Females |
| SNB angle | Angle between the Sella, Nasion and B point in a cephalogram | 81.6 ± 3.8 | 81.1 ± 3.9 |
| ANB angle | Angle between A point, Nasion and B point in a cephalogram | 3.8 ± 2.2 | 3.9 ± 2.2 |
| Incisor overjet | Amount of horizontal overlap of the maxillary central incisors over the mandibular central incisors | 3.5 ± 2.7 | 3.8 ± 4.1 |
| Wits appraisal | Distance between perpendiculars drawn from points A and B on the maxilla and the mandible, respectively to the occlusal plane | 1.2 ± 1.9 | -0.1 ± 1.8 |
