# Supplementary material for: The Oropharyngeal Airway in Young Adults with Skeletal Class II and Class III Deformities: A 3-D Morphometric Analysis
Source: PLoS One. 2016 Feb 22;11(2):e0148086. doi: 10.1371/journal.pone.0148086 (PMC4762707; doi:10.1371/journal.pone.0148086)
Supplement: S2 Appendix — (DOCX) [file pone.0148086.s002.docx]

S2 Appendix. The technical error of measurement (TEM) for different oropharyngeal parameters

| Measurement | TEM |
| --- | --- |
| RP Space length (mm) | 1.44 |
| RG Space length (mm) | 1.35 |
| RG Volume (mm^3^) | 0.36 |
| Level of most constricted RG area | 0.25 |
| RG Minimum cross sectional area (mm^2^) | 2.18 |
| Width of the most constricted RG area (mm) | 1.48 |
| AP length of the most constricted RG area (mm) | 0.38 |
| RP Volume (mm^3^) | 0.90 |
| Level of most constricted RP area | 0.09 |
| RP Minimum cross sectional area (mm^2^) | 2.24 |
| Width of the most constricted RP area (mm) | 1.19 |
| AP length of the most constricted RP area (mm) | 1.76 |
